# Pyroptosis Patterns Characterized by Distinct Tumor Microenvironment Infiltration Landscapes in Gastric Cancer

**DOI:** 10.3390/genes12101535

**Published:** 2021-09-28

**Authors:** Renshen Xiang, Yuhang Ge, Wei Song, Jun Ren, Can Kong, Tao Fu

**Affiliations:** Department of Gastrointestinal Surgery II, Renmin Hospital of Wuhan University, Wuhan 430060, China; 2014302180192@whu.edu.cn (R.X.); 2013302180234@whu.edu.cn (Y.G.); whdxsw@whu.edu.cn (W.S.); renjun0414@163.com (J.R.); kong_can@163.com (C.K.)

**Keywords:** gastric cancer, pyroptosis, tumor microenvironment, genetic variation, immunotherapy

## Abstract

Background: The potential role of pyroptosis in tumor microenvironment (TME) reprogramming and immunotherapy has received increasing attention. As most studies have concentrated on a single TME cell type or a single pyroptosis regulator (PR), the overall TME cell-infiltrating characteristics mediated by the integrated roles of multiple PRs have not been comprehensively recognized. Methods: This study curated 33 PRs and conducted consensus clustering to identify distinct pyroptosis patterns in gastric cancer (GC) patients. A single-sample gene set enrichment analysis algorithm was used to quantify the infiltration density of TME immune cells and the enrichment scores of well-defined biological signatures. The pyroptosis patterns of individuals were quantified using a principal component analysis algorithm called the pyroptosis score (PS). Results: Three distinct pyroptosis patterns with significant survival differences were identified from 1422 GC samples; these patterns were closely associated with three TME cell-infiltrating landscapes—namely, the immune-inflamed, immune-excluded, and immune-desert phenotypes. The PS model generated on the basis of the pyroptosis pattern-related signature genes could accurately predict the TME status, existing molecular subtypes, genetic variation, therapeutic response, and clinical outcome; among which, a relatively high PS was highly consistent with immune activation, molecular subtypes with survival advantages, high tumor mutation burden, high microsatellite instability, and other favorable characteristics. In particular, from the Cancer Genome Atlas database, the PS model exhibited significant prognostic relevance in a pan-cancer analysis, and patients with a relatively high PS exhibited durable therapeutic advantages and better prognostic benefits in anti-PD1/L1 therapy. Conclusions: This study demonstrates that pyroptosis is prominently correlated with TME diversity and complexity, and quantification of the pyroptosis patterns of individuals will enhance our cognition of TME infiltration landscapes and help in formulating more effective immunotherapeutic strategies.

## 1. Introduction

Immunotherapy, including approved immune checkpoint inhibitors (ICIs), anti-cytotoxic T-lymphocyte-associated protein 4 (anti-CTLA-4), anti-programmed cell death-1/programmed cell death-Ligand 1 (anti-PD-1/PD-L1) antibody and chimeric antigen receptor (CAR) T-cell therapy, has achieved considerably improved clinical outcomes in cancer treatment [1,2]. However, only a small proportion of patients have achieved durable benefits [3], which are closely related to PD-1/PD-L1 expression, microsatellite status, tumor mutation burden (TMB), and the tumor microenvironment (TME). Traditionally, the malignant progression of cancer is a multistep process involving genetic and epigenetic variations in tumor cells. However, several studies have shown that the TME of tumor cell growth and survival plays a crucial role in tumor progression. The TME is composed of heterogeneous populations, including tumor cells, blood vessels, infiltrating immune cells, stromal cells (e.g., fibroblasts), tissue fluid, and cytokines [4]. Tumor cells are equivalent to “seeds”, whereas TME is similar to “soil”. The two constantly interact to inhibit apoptosis; avoid hypoxia; and induce cell proliferation, angiogenesis, and immune tolerance. Therefore, the occurrence, invasion, metastasis, and drug resistance of tumors are inseparable from the TME [4]. Tumor cells can be recognized, monitored, and eliminated through cytotoxic lymphocytes in immune-activated TME, while they can escape in an immunosuppressive state. Antitumor immune cells are composed of effector T cells, natural killer cells, dendritic cells, M1-type macrophages, and N1-type tumor-associated neutrophiles (TANs), whereas inhibitory TME is associated with regulatory T cells, marrow-derived suppressor cells (MDSCs), N2-type TANs, tumor-associated fibroblasts (CAFs), and M2-type macrophages [4]. Accordingly, a comprehensive analysis of the heterogeneity and complexity of TME can identify distinct tumor immunophenotypes and improve the predictive ability of immunotherapy.

Cell death is one of the most fundamental issues in life. However, because of their inherent genetic and epigenetic heterogeneity and metabolic plasticity, tumor cells are highly adaptable to adverse TME [5]. Cell death can be divided into necrosis and programmed cell death (PCD). Apoptosis is a type of PCD that is caused by the self-destruction of cells controlled by genes. The membrane of apoptotic cells remains intact and limits the inflammatory response; thus, it remains silent in terms of immunogenicity. Necrosis is passive cell death caused by pathological stimuli. The increased permeability of necrotic cells causes cell swelling, cell rupture, and the release of cell contents, which leads to an inflammatory reaction and is highly immunogenic [6]. Pyroptosis is an inflammatory cell death usually caused by microbial infection, accompanied by the activation of inflammasomes and the maturation of proinflammatory interleukin-1β (IL-1β) and interleukin-18 (IL-18) [7]. Gasdermins are a family of intracellular proteins that execute pyroptosis. The cytotoxic N-terminal of gasdermins generated from caspases or the granzyme protease-mediated cleavage of gasdermin proteins oligomerizes and forms pores across the cell membrane, leading to the release of IL-1β and IL-18 [7]. Similar to apoptosis, pyroptosis can also cause nuclear condensation and chromatin DNA fragmentation [8,9]. Moreover, similar to the case of necrosis, the formation of pores across the cell membrane during pyroptosis disrupts the balance of ion gradients inside and outside the cell membrane, which causes the extracellular fluid to flow into the cell, thereby leading to cell swelling, cell rupture, and the release of proinflammatory factors, such as IL-1β and IL-18 [10].

With the advancement of research, increasing evidence has demonstrated that pyroptosis reprograms TME and is associated with the tumor outcome [7,11]. On one hand, the activation of multiple signal pathways and the release of inflammatory mediators during pyroptosis are closely related to malignant tumor progression and drug resistance [11]. On the other hand, pyroptosis, as a type of cell death, can kill tumor cells by itself. Moreover, pyroptosis is highly immunogenic and can recruit inflammatory cell infiltration and cause local inflammation, thereby alleviating immunosuppression and inducing the immune response of the TME [7,11]. Consequently, pyroptosis can provide a novel therapeutic strategy for tumors, and comprehensively recognizing the TME cell infiltration characterizations mediated by pyroptosis can contribute to enhancing our understanding of TME immune regulation. Therefore, this study focuses on the effect of pyroptosis on TME reprogramming in gastric cancer (GC) patients and briefly explores its role in pan-cancer analysis.

## 2. Materials and Methods

### 2.1. Acquisition and Processing of Publicly Attainable Expression Datasets

The workflow of this study is shown in Figure S1. Gene expression data and complete clinical traits were retrieved from the Gene Expression Omnibus (GEO) and Cancer Genome Atlas (TCGA) databases. Patients with a survival time of < 30 days and those with an ambiguous survival status were excluded. Six eligible GC cohorts (namely, GSE84437, GSE57303, and GSE62254 for ACRG typing; GSE15459 for Lei typing; GSE34942 for Lei typing; and TCGA-STAD) were collected for the subsequent analysis. In the TCGA cohort, the fragment per kilobase million (FPKM) values of the gene expression were accessed from the TCGA portal (http://cancergenome.nih.gov) on 14 February 2021. The FPKM values were then converted into transcripts per kilobyte (TPM) values. From the microarray data obtained from the GEO database (https://www.ncbi.nlm.nih.gov/geo/ on 14 February 2021), the normalized RNA sequencing data were directly obtained for further analysis. The ComBat algorithm from the “SVA” package was used to remove the batch effects from nonbiological technical biases among the six datasets. The somatic mutation data and copy number variation (CNV) data were accessed from the TCGA database. The clinical traits of the meta-cohort are listed in Table S1.

### 2.2. Unsupervised Clustering for Pyroptosis Regulators

In all, 33 pyroptosis regulators (PRs) were identified from the literature [11,12,13,14] (Table S2). Nevertheless, only 32 PRs were extracted from a meta-cohort composed of five GEO datasets and a TCGA cohort to identify distinct pyroptosis patterns through an unsupervised clustering analysis. The number of clusters and their stability were determined using a consensus clustering algorithm, which was executed using the “ConsensuClusterPlus” package and repeated 1000 times [15].

### 2.3. Gene Set Variation Analysis and Functional Annotation

The “c2.cp.kegg.v6.2.symbols” package [16] was downloaded from the MSigDB database for carrying out a gene set variation analysis (GSVA), which was executed to investigate the difference in the molecular biological pathways among the distinct pyroptosis patterns using the “GSVA” package [17]. The “clusterProfiler” package was used to perform the Kyoto Encyclopedia of Genes and Genomes (KEGG) enrichment analysis with a cutoff value of <0.05 for the false discovery rate (FDR).

### 2.4. Estimation of TME Status, TME Cell Infiltration, and Well-Defined Biological Signatures

The immune/stromal scores and tumor purity for each sample were calculated using the “ESTIMATE” package. The marker genes for the immune cell type were accessed from Charoentong’s paper [18], and the infiltrating fraction of each cell was quantified using the single-sample gene set enrichment analysis (ssGSEA) algorithm. Well-defined biological signatures were derived from the gene sets developed by Hallmarker [16] and Mariathasan et al. [19]. The enrichment fraction estimated using ssGSEA represented the relative activity of the biological pathways in the GC samples.

### 2.5. Identification of Differentially Expressed Genes between Distinct Pyroptosis Patterns

Under the criterion of FDR < 0.01, the “limma” package was used for identifying differentially expressed genes (DEGs) across distinct pyroptosis patterns [20]. In particular, the gene expression data were normalized by Vom and then input into the lmFit and eBayes functions to evaluate the differential expression statistics.

### 2.6. Quantification of Pyroptosis Patterns by Using the Principal Component Analysis Algorithm

A set of scoring systems was constructed to evaluate the pyroptosis patterns of individuals by using the principal component analysis (PCA) algorithm; this system was named the pyroptosis score (PS). In particular, consensus clustering was performed on GC patients according to the expression of overlapping DEGs. Next, a Cox regression model was conducted for each overlapping DEG. The prognostic DEGs (*p* < 0.05) were enrolled in PCA for generating the pyroptosis-related gene signature, and principal components 1 and 2 were extracted as the signature scores. This approach focused on the score of the set with the largest block of well-correlated or inversely correlated genes in the set while downweighing the contribution from genes that did not track the other set members. Finally, a formula similar to that used in previous studies was used to define the PS [21]: PS = ∑(PC1_i_ + PC2_i_), where “i” represents the expression of pyroptosis pattern-related genes. Subsequently, a correlation analysis was conducted to determine the association between the PS and TME cell infiltration and well-defined biological signatures.

### 2.7. Sensitivity of Chemotherapeutic Agents

The “pRRophetic” package was utilized for predicting the chemotherapeutic response of GC samples to 16 agents (namely, camptothecin, methotrexate, mitomycin C, doxorubicin, gemcitabine, paclitaxel, imatinib, blemycin, docetaxel, sunitinib, cisplatin, vinblastine, sorafenib, tipifarnib, temsirolimus, and axitinib) on the basis of the Genomics of Drug Sensitivity in Cancer (GDSC) database (https://www.cancerrxgene.org/ on 14 February 2021). The half-maximum inhibitory concentration (IC_50_) of the sample was estimated using ridge regression, and a 10-fold cross-validation was used to evaluate the prediction accuracy on the basis of the GDSC training set.

### 2.8. Collection of Genomic and Clinical Information from ICI Therapy-Based Cohorts

We systematically searched for immunotherapeutic cohorts with publicly available gene expression profiles and clinical traits. Two ICI therapeutic cohorts were finally enrolled in our study: the GSE78220 cohort treated with pembrolizumab (anti-PD-1) for metastatic melanoma [22] and the IMvigor210 cohort treated with atezolizumab (anti-PD-L1) for advanced urothelial carcinoma [19]. The gene expression data of the biopsy samples before treatment were collated and converted into TPM values for the subsequent analysis.

### 2.9. Application of the PS Model to Pan-Cancer Analysis

The gene expression profiles and survival information of various tumors, such as esophageal carcinoma, cholangiocarcinoma, liver hepatocellular carcinoma, pancreatic adenocarcinoma, colon adenocarcinoma, rectum adenocarcinoma, glioblastoma multiforme, lower-grade glioma, adrenocortical carcinoma, bladder urothelial carcinoma, kidney chromophobe, kidney renal clear cell carcinoma, kidney renal papillary cell carcinoma, pheochromocytoma, paraganglioma, testicular germ cell tumor, prostate adenocarcinoma, acute myeloid leukemia, diffuse large B-cell lymphoma, lung squamous cell carcinoma, thymoma, uveal melanoma, breast invasive carcinoma, cervical squamous cell carcinoma, endocervical adenocarcinoma, uterine corpus endometrial carcinoma, uterine carcinosarcoma, and ovarian serous cystadenocarcinoma, were downloaded from the TCGA database (http://cancergenome.nih.gov on 14 February 2021). The PS of each tumor sample was calculated using the PCA algorithm, and the prognostic value of the PS was evaluated using Kaplan–Meier analysis and a log–rank test.

### 2.10. Statistical Analysis

All data were analyzed with Strawberry Perl software (64-bit), R software (version 4.0.5), and Bioconductor packages. The genetic variation of PRs and the mutation landscape in the high-and low-PS patient subgroups were determined using the waterfall function of the “maftools” package. The “RCircos” package was used for drawing the CNV landscape of PRs in 23 pairs of chromosomes [23]. A univariate analysis was used to calculate the hazard ratios (HRs) of the factors, a multivariate analysis was used to determine their independent prognostic factors, and the results were visualized using the “forestplot” package. The Kaplan–Meier analysis and the log-rank test were used for evaluating the overall survival (OS) or disease-free survival (DFS). The surv_cutpoint function from the “survival” package determined the cutoff point of the PS in each dataset. The area under the receiver operating characteristic (ROC) curve for evaluating the specificity and sensitivity was calculated using the “survivalROC” package. For the quantitative data, the normally distributed variables were analyzed using a Student’s *t*-test, whereas the non-normally distributed variables were estimated using the Wilcoxon rank-sum test. For comparisons of more than two groups, the one-way analysis of variance and Kruskal–Wallis tests were used as the parametric and nonparametric methods, respectively [24]. All comparisons were two-sided, with *p* < 0.05 denoting statistical significance.

## 3. Results

### 3.1. Landscape of Genetic Variations of PRs in GC

**Figure 1:**
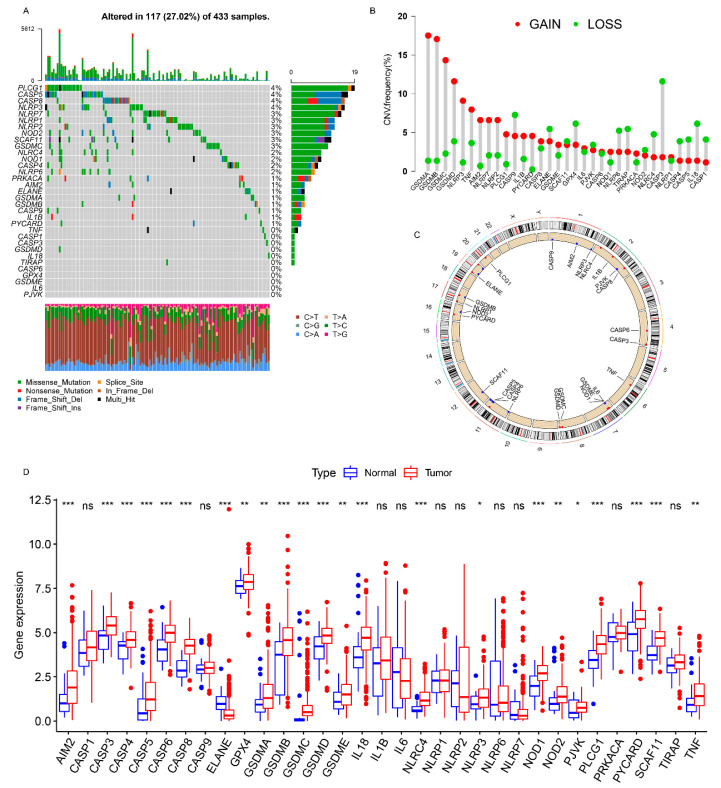
Landscape of genetic alterations of PRs in GC. (**A**) The mutation frequency of PRs in 433 patients with GC. The number on the right represented the mutation frequency of the PRs. Each column corresponds to one of the 117 TCGA samples (out of the 433) where there was at least one genetic alteration in the PRs. (**B**) The CNV mutation frequency of the PRs. The column showed the alteration frequency. The amplification frequency, red dot; the deletion frequency, green dot. (**C**) The location of the CNV alteration of the PRs on the chromosomes. (**D**) Differential expression analysis of the PRs between normal tissues and GC tissues. * *p* < 0.05; ** *p* < 0.01; *** *p* < 0.001.

The landscape of the genetic variations (somatic mutation and CNV) of 33 PRs in GC was explored using the TCGA cohort. The KEGG enrichment analysis showed that PRs were involved in the regulation of inflammatory or immune-related pathways (e.g., positive regulation of cytokine production, interleukin-1 production, execution phase of apoptosis, and positive regulation of interleukin-8 production) (Figure S2A). Among the 433 samples, 117 (27.02%) experienced genetic alterations of PRs, primarily including missense mutation, splice site, and nonsense mutation. *PLCG1*, *CASP5*, *CASP8*, and *NLPR3* showed the highest mutation frequency, followed by *NLPR7*, *GSDMC*, *SCAF11*, *NOD2*, *NLRP2*, and *NLRP1*, whereas *TNF*, *PJVK*, *IL6*, *GSDME*, *GPX4*, *CASP6*, *TIRAP*, *IL18*, *GSDMD*, *CASP3*, and *CASP1* did not mutate in the GC samples (Figure 1A). We also found a significant co-mutation relationship between *NLRP6* and *CASP5*, as well as that between *NOD1* and *NLRC4* (Figure S2B). A further analysis demonstrated that genetic mutations in *NLRP3* and *PLCG1* significantly altered their expression levels (Figure S3A–N), and the higher the mutation frequency, the more significant was the change in the expression level. The investigation of the CNV alteration frequency revealed a widespread CNV alteration in 33 PRs, 19 of which focused on copy number amplification, while the other 14 concentrated on copy number deletion (Figure 1B). The locations of CNV alterations of PRs on a chromosome are shown in Figure 1C. Moreover, the differential expression analysis revealed that PRs with obvious amplified/deleted CNVs (e.g., *AIM2* and *GSDMA*) were significantly altered between the normal and GC tissues (Figure 1B,D), which demonstrated that CNV alterations can be the prominent factors resulting in perturbations in the PR expression. A univariate Cox regression analysis demonstrated that *TIRAP*, *CASP8*, *GSDMD*, *CASP6*, *CASP3*, *CASP5*, *GSDMC*, *CASP1*, *NLRP6*, *GSDMB*, and *AIM2* could be considered protective factors, while *NLRP3*, *PLCG1*, *PJVK*, *IL6*, *GSDME*, and *GPX4* were recognized as risk factors. Furthermore, a multivariate Cox regression analysis revealed that *TIRAP*, *CASP1*, and *GSDME* were three independent prognostic factors (Figure S2D). The above analyses proved the genetic and transcriptomic heterogeneity in PRs between the normal and GC samples and revealed a close connection between the genetic variation and the expression alterations. Therefore, the genomic and transcriptomic landscape in PRs plays an indispensable role in regulating GC occurrence and progression.

### 3.2. Identification of Pyroptosis Patterns Based on a Meta-Cohort

**Figure 2:**
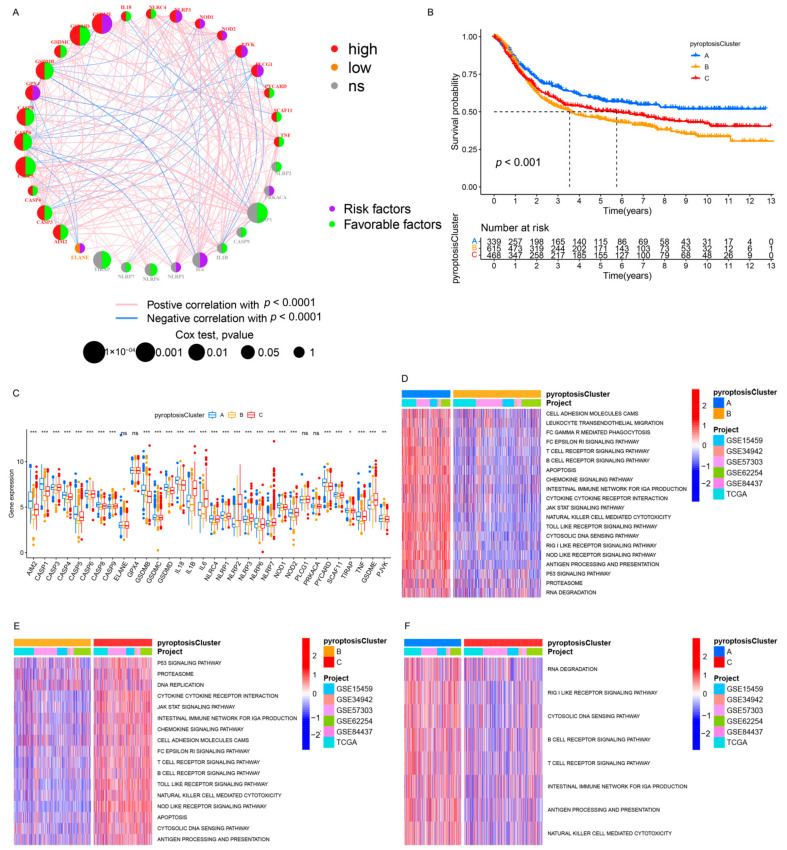
Pyroptosis patterns and relevant biological pathway. (**A**) The interaction between PRs in GC. The circle size indicated the effect of PRs on the prognosis, and the range of values calculated by the Cox test was *p* < 1 × 10^−4^, *p* < 0.001, *p* < 0.01, *p* < 0.05, and *p* < 0.1, respectively. Purple semicircle on the right, risk factors; green semicircle on the right, favorable factors. Red semicircle on the left, upregulated PRs in GC; orange semicircle on the left, downregulated PRs in GC; gray semicircle on the left, no difference in expression. The lines connecting PRs represented their interactions, and the thickness represented the correlation strength between PRs. The blue line marked a negative correlation, and the red line marked a positive correlation. (**B**) Kaplan–Meier analysis of the OS for 1422 GC patients in the meta-cohort with different pyroptosis patterns (Log-rank test: *p* < 0.001). (**C**) Differential expression analysis of PRs among three pyroptosis patterns. (**D**–**F**) GSVA enrichment analysis. The heatmap was used for visualizing pathways, and red indicated activated pathways and blue indicated inhibited pathways. (**D**) Pyroptosis cluster A vs. pyroptosis cluster B. (**E**) Pyroptosis cluster B vs. pyroptosis cluster C. (**F**) Pyroptosis cluster A vs. pyroptosis cluster C. * *p* < 0.05; ** *p* < 0.01; *** *p* < 0.001.

The TCGA cohort and five GEO datasets (the GSE84437, GSE15459, GSE34942, GSE57303, and GSE62254/ACRG cohorts), which contained complete clinical information and survival annotations, were integrated into a meta-cohort. A comprehensive landscape of the expression levels of the PRs, the correlation connections, and their prognostic relevance in GC patients was illustrated in the network (Figure 2A and Table S3). These results indicated that cross-talk between PRs plays a critical role in the formation of pyroptosis patterns among individuals. Accordingly, unsupervised clustering was used to stratify GC patients with qualitatively distinct pyroptosis patterns on the basis of the expression of PRs, and three stable patterns were identified, with 339 samples in cluster A, 615 samples in cluster B, and 468 samples in cluster C (Figure 2B and Figure S4A and Table S4). These patterns were termed pyroptosis clusters A, B, and C, respectively; among which, cluster A showed a remarkable survival advantage, followed by cluster C, whereas cluster B exhibited the worst OS in the meta-cohort (*p* < 0.001, Figure 2B). Furthermore, we observed significant differences in the expression of PRs between distinct pyroptosis patterns, as most PRs (e.g., *NOD2* and *SCAF11*) were downregulated in pyroptosis cluster B and elevated in pyroptosis clusters A and C (Figure 2C and Figure S4B).

### 3.3. Pyroptosis Patterns Characterized by Distinct TME Infiltration Landscapes

**Figure 3:**
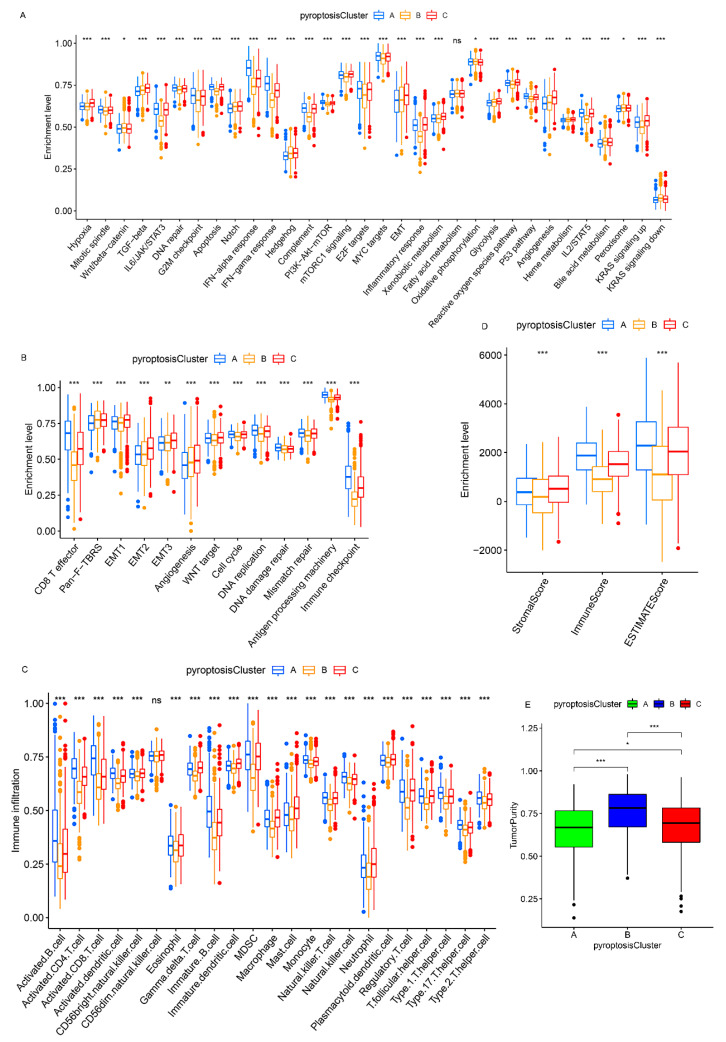
TME characteristics and enrichment levels of well-defined biological signatures in distinct pyroptosis patterns. (**A**,**B**) Three pyroptosis patterns were distinguished by Hallmark pathways curated from the MSigDB database (**A**), and Mariathasan et al. constructed gene sets (**B**) in the meta-cohort. (**C**) The abundance of TME-infiltrating cells in distinct pyroptosis patterns. (**D**) Stromal/immune scores in three patterns. (**E**) Tumor purity in three patterns. * *p* < 0.05; ** *p* < 0.01; *** *p* < 0.001.

A subsequent analysis was performed to explore the molecular biological behaviors across the three pyroptosis patterns. GSVA demonstrated that pyroptosis cluster A was markedly enriched in immune activation-related pathways, such as the T-cell receptor signaling pathway, B-cell receptor signaling pathway, and the natural killer cell-mediated cytotoxicity (Figure 2D,F); pyroptosis cluster B was closely associated with immune suppression processes (Figure 2D,E); and the enrichment fraction of the immune pathways in pyroptosis cluster C was between that of pyroptosis cluster A and that of pyroptosis cluster B (Figure 2E,F). Moreover, ssGSEA revealed that pyroptosis cluster A maintained its immune-inflamed properties (e.g., IFN-alpha response, IFN-gamma response, and CD8 T effector); pyroptosis cluster B exhibited a complete immune desert state with partial stromal components (e.g., notch signal and pan-F-TBRS); and pyroptosis cluster C was prominently related to carcinogenic activation and stromal pathways (e.g., hypoxia, EMT, and angiogenesis), in addition to a certain degree of relation to the immune activation pathways (Figure 3A,B and Figure S4C). A further analysis indicated that pyroptosis cluster A exhibited the lowest tumor purity, the highest immune score, and abundant antitumor lymphocyte cell subpopulations (e.g., activated B cells, activated CD4+ T cells, and activated CD8+ T cells); pyroptosis cluster B retained the highest tumor purity and the lowest immune/stromal scores and immune cells; and like pyroptosis cluster A, pyroptosis cluster C exhibited the highest stromal score along with the immune cells (Figure 3C–E and Figure S4D). In summary, the three pyroptosis patterns exhibited remarkably distinct TME infiltration characterizations. Pyroptosis cluster A was defined as an immune-inflamed phenotype characterized by immune cell infiltration and immune activation, pyroptosis cluster B was classified as an immune-desert phenotype characterized by complete immunosuppression and partial stromal activation, and pyroptosis cluster C was recognized as an immune-excluded phenotype characterized by stromal activation and weakened immune cell infiltration.

Next, we investigated the connection between PRs and immune cell infiltration using Spearman’s correlation analysis. The upregulation of *AIM2*, *CASP1*, *CASP4*, *IL1B*, *IL6*, *NLRC4*, and *NLRP3* was positively correlated with the enhancement of immune cell infiltration, whereas the overexpression of *CASP6*, *CASP9*, *PLCG1*, *TIRAP*, and *PJVK* was negatively associated with immune cell infiltration (Figure S5A). Among these PRs, *AIM2* attracted our attention because of its positive correlation to the immune score (Figure S5B), abundant lymphotoxic cells (Figure S5C), increased MHC molecules (Figure S5D), and immune response activation (Figure S5E). Upregulated *AIM2* predicted better survival outcomes (*p* < 0.001, Figure S5F). Note that AIM2 can activate inflammasomes in GC patients and act as a tumor suppressor by inducing IL-18 and IL-1β [25], indicating that *AIM2* is an important prognostic indicator in GC patients, which is consistent with our study.

### 3.4. Pyroptosis Patterns in GSE62254/ACRG Cohort

**Figure 4:**
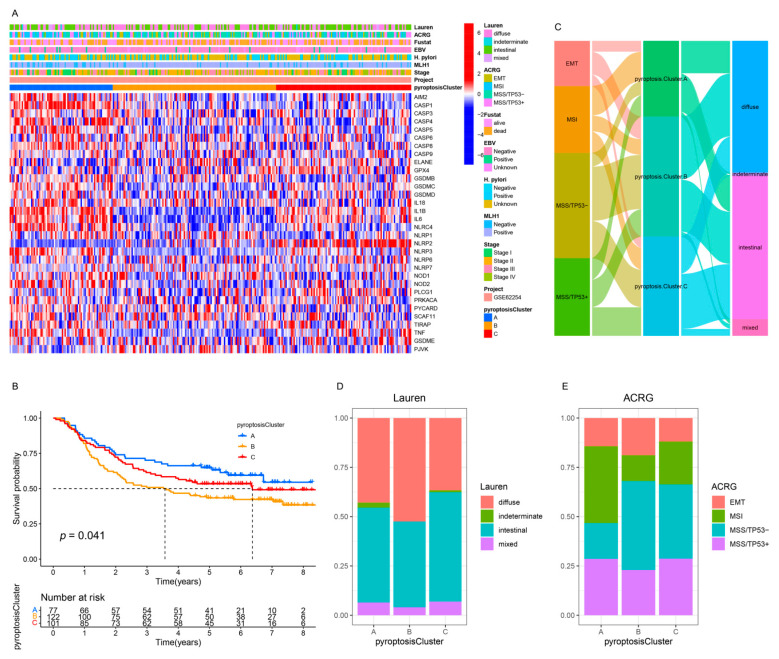
Integrated analysis of three pyroptosis patterns in the GSE62254/ACRG cohort. (**A**) The heatmap of the expression of the PRs and clinicopathologic traits in distinct pyroptosis patterns. (**B**) Kaplan–Meier analysis of the OS for GC patients with distinct pyroptosis patterns (Log-rank test: *p* = 0.041). (**C**) Alluvial diagram of pyroptosis patterns in groups with different molecular subtypes. (**D**,**E**) The proportion of Lauren subtypes (**D**) and ACRG subtypes (**E**) in the three pyroptosis patterns.

To further explore the relationship among the three pyroptosis patterns and the clinicopathologic characteristics and existing GC subtypes, we focused on the GSE62254/ACRG cohort, which exhibited comprehensive clinical traits. As in the meta-cohort, unsupervised clustering identified three distinct pyroptosis patterns in the GSE62254/ACRG cohort (Figure S6A–D and Figure 4A). The pyroptosis clusters A and C were characterized by the upregulation of PRs, whereas pyroptosis cluster B was characterized by the downregulation of PRs (Figure S6E and Figure 4A), which was consistent with the meta-cohort (Figure S4B and Figure 2C). The survival results of the three patterns (*p* = 0.041, Figure 4B) also showed the same outcomes as those observed for the meta-cohort (*p* < 0.001, Figure 2B). Further analyses demonstrated that patients with the diffuse histological subtype (Figure 4C,D) and the EMT and MSS/TP53 molecular subtypes (Figure 4C,E) were mainly concentrated in the pyroptosis cluster B pattern, whereas those with the MSI subtypes were mostly enriched in the pyroptosis cluster A pattern (Figure 4C,E). Moreover, patients with negative EBV and/or helicobacter pylori (*H. pylori*) infection (Figure S6F,G); advanced clinicopathologic traits (e.g., lymphovascular invasion, venous invasion, perineural invasion, upregulated MLH1, T3–4, N2–3, M1, and stage III–IV) (Figure S6H–O); and recurrence sites (e.g., ascites and peritoneum) (Figure S6P–T) were characterized by the pyroptosis cluster B pattern, whereas pyroptosis cluster A was relevant to the best clinicopathologic outcomes (Figure S6F–T). Consistent with the meta-cohort, the GSE62254/ACRG cohort confirmed that patients characterized by the pyroptosis cluster A pattern correlated with immune activation, whereas the tumors characterized by the pyroptosis cluster A pattern were associated with malignant progression.

### 3.5. Generation of Pyroptosis Pattern-Related Signature Genes and Functional Annotation

**Figure 5:**
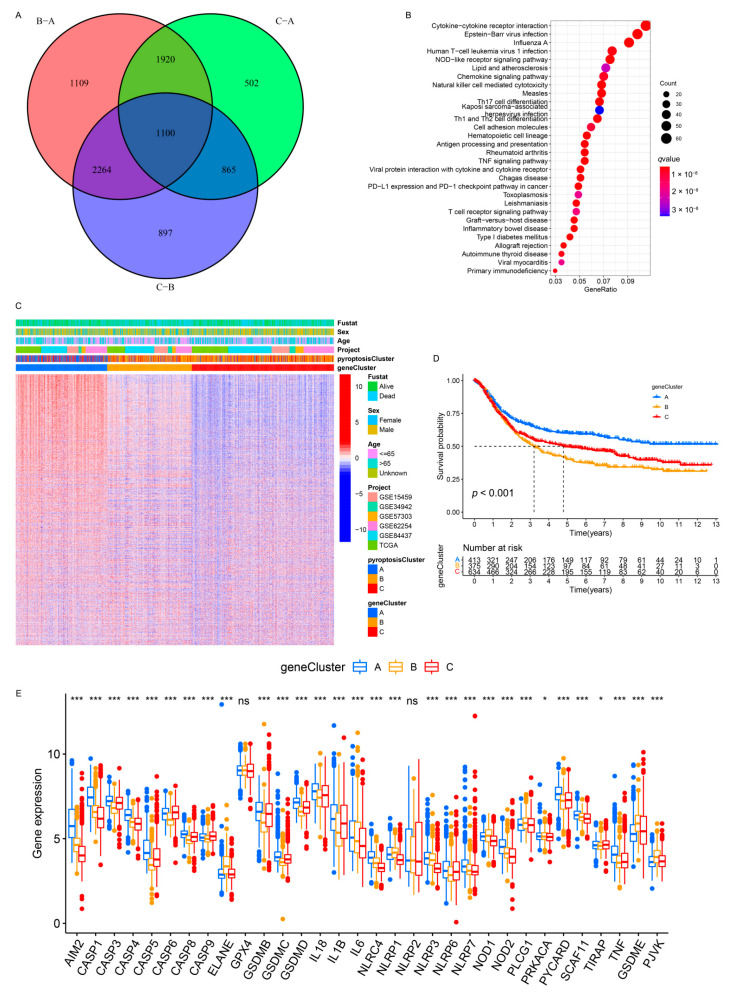
Unsupervised clustering of pyroptosis pattern-related genes in the meta-cohort. (**A**) One thousand and one hundred pyroptosis pattern-related genes were shown in the Venn diagram. (**B**) KEGG enrichment analysis for pyroptosis pattern-related genes. The x-axis represented the gene ratio. (**C**) Unsupervised clustering of overlapping pyroptosis pattern-related genes in the meta-cohort divided the patients into three distinct clusters, called gene clusters A–C, respectively. (**D**) Kaplan–Meier analysis for three distinct gene clusters with 1422 GC patients in the meta-cohort (Log-rank test: *p* < 0.001). (**E**) The expression of the PRs in the three gene clusters in the meta-cohort. The upper and lower ends of the boxes represented an interquartile range of the values. The lines in the boxes represented the median value (one-way ANOVA test: * *p* < 0.05; *** *p* < 0.001).

**Figure 6:**
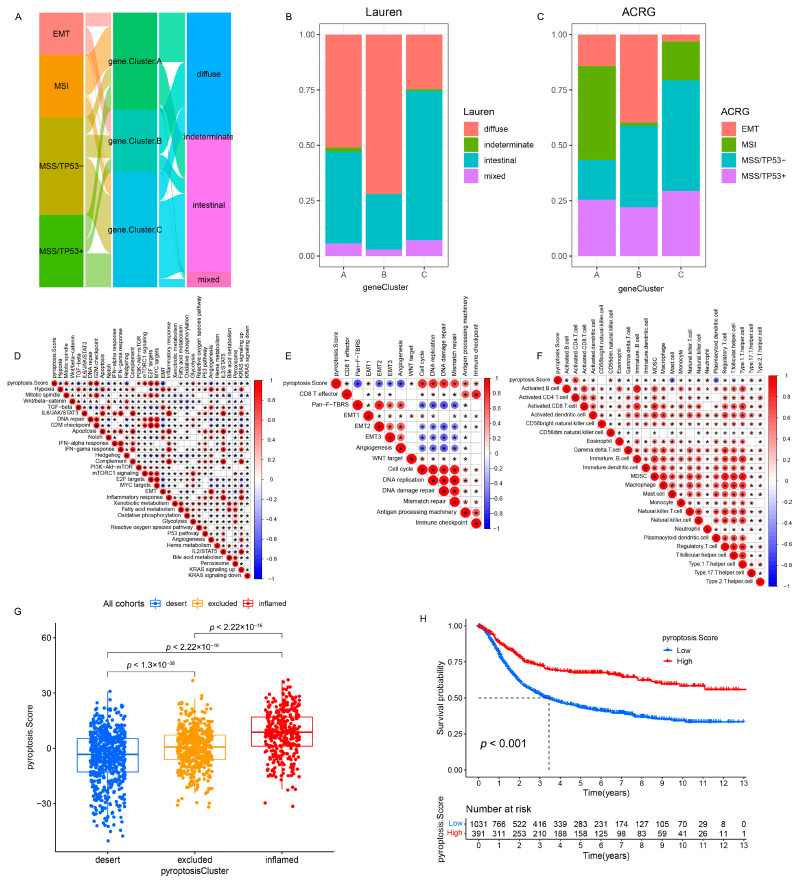
Correlation between the PS and TME status and relevant biological signatures in the meta-cohort. (**A**) Alluvial diagram of gene clusters in groups with different ACRG subtypes and Lauren subtypes in the GSE62254/ACRG cohort. (**B**) The proportion of Lauren subtypes in three gene clusters in the GSE62254/ACRG cohort. (**C**) The proportion of ACRG subtypes in three gene clusters in the GSE62254/ACRG cohort. (**D**–**F**) The correlation between the PS and Hallmark pathways (**D**), Mariathasan et al. constructed gene sets (**E**), and TME infiltration cells (**F**) using a Spearman analysis in the meta-cohort. A negative correlation was marked with blue and a positive correlation with red. *p* < 0.05. (**G**) Differences in the PS among the three phenotypes in the meta-cohort (*p* < 0.001, Kruskal–Wallis test). (**H**) Kaplan–Meier analysis for patients with high and low PS subgroups in the meta-cohort (Log-rank test: *p* < 0.001).

To further investigate the potential genetic alterations and/or expression perturbations within the three patterns, we identified 1100 pyroptosis phenotype-related overlapping DEGs using the limma package (Figure 5A and Table S5). The KEGG enrichment analysis of the DEGs revealed that the immunoregulatory pathways (e.g., natural killer cell-mediated cytotoxicity, Th17 cell differentiation, Th1 and Th2 cell differentiation, T-cell receptor signaling pathway, and PD-L1 expression and PD-1 checkpoint pathway in cancer) were significantly over-presented (Figure 5B), which was extremely similar to the functionality of the PRs (Figure S2A). These results indicate that the overlapped DEGs were remarkably associated with pyroptosis and could be called the most representative pyroptosis pattern-related signature genes (PPRSGs). To validate the existence of the pyroptosis patterns, we conducted unsupervised clustering using PPRSGs. Consistent with the results of the consensus clustering for the PRs, the PPRSGs identified three distinct pyroptosis phenotypes in the meta-cohort (Figure S7A–D) and the GSE62254/ACRG cohort (Figure S7E–H), which were defined as gene clusters A, B, and C, respectively, characterized by different signature genes (meta-cohort: Figure 5C; ACRG cohort: Figure S7I). Gene cluster A had the best OS, gene cluster C had the second-best OS, and gene cluster B had the worst OS (meta-cohort: Figure 5D; ACRG cohort: Figure S7J). Moreover, the expression of PRs in gene clusters A–C (meta-cohort: Figure 5E; ACRG cohort: Figure S7K) was identical to that of PRs in pyroptosis clusters A–C (meta-cohort: Figure 2C and Figure S4B; ACRG cohort: Figure 4A and Figure S6E): PRs were upregulated in gene clusters A and C and pyroptosis clusters A and C; they were downregulated in gene cluster B and pyroptosis cluster B. More importantly, patients with MSI subtypes were mostly concentrated in gene cluster A (Figure 6A,C), whereas those with EMT molecular subtypes and diffuse histological subtypes were characterized by the gene cluster B phenotype (Figure 6A–C), which was in accordance with the expected results of the pyroptosis patterns (Figure 4C–E). These analyses revealed three distinct pyroptosis patterns in the GC patients.

### 3.6. Quantification of Pyroptosis Patterns and Their Relationship with Existing GC Typing

**Figure 7:**
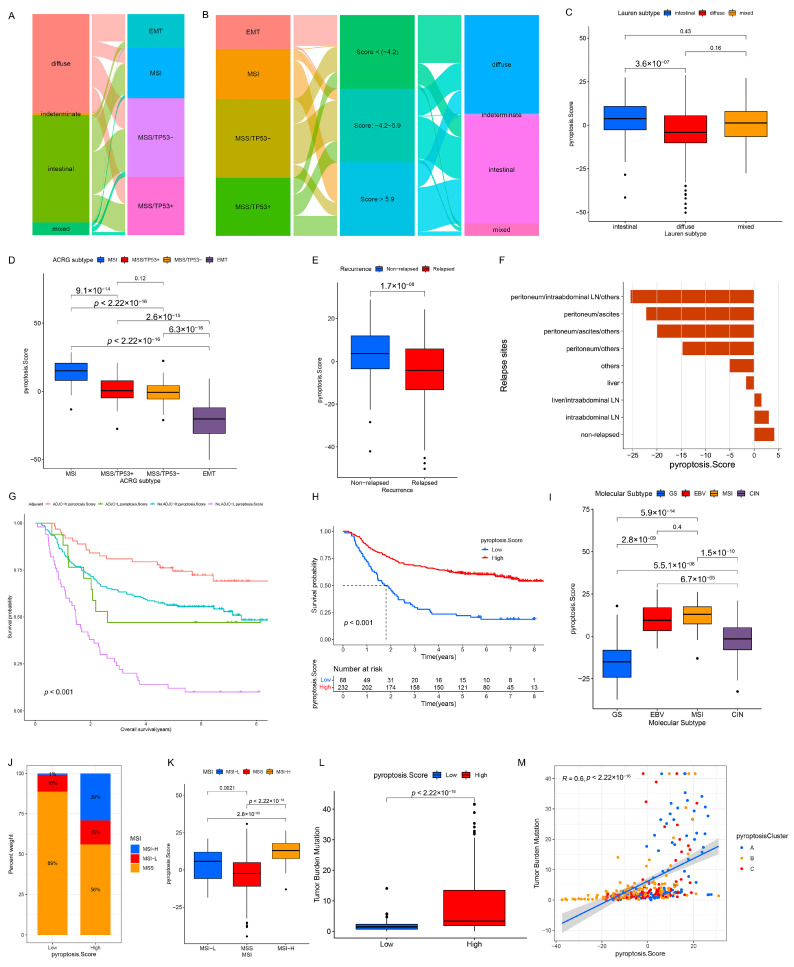
Correlation between the PS and existing GC subtypes and clinicopathologic traits. (**A**) Alluvial diagram of Lauren subtypes in groups with different ACRG subtypes in the GSE62254/ACRG cohort. (**B**) Alluvial diagram of the PS subgroups in groups with different ACRG subtypes and Lauren subtypes in the GSE62254/ACRG cohort. (**C**–**E**) Differences in the PS among Lauren subtypes (**C**), ACRG subtypes (**D**), and relapsed (or non-relapsed) subgroups (**E**) in the GSE62254/ACRG cohort (Kruskal–Wallis test). (**F**) The PS in different recurrent sites in the GSE62254/ACRG cohort. (**G**) Overall survival analysis for the subgroup patients stratified by both the PS and treatment with adjuvant chemotherapy using Kaplan–Meier curves. H, high; L, Low; ADJC, adjuvant chemotherapy (Log-rank test: *p* < 0.001). (**H**) Kaplan–Meier analysis for patients with high- and low-PS subgroups in the GSE62254/ACRG cohort (Log-rank test: *p* < 0.001). (**I**) Differences in the PS among the TCGA subtypes in the TCGA cohort. (**J**) The proportion of patients with different MSI statuses in the PS subgroups in the TCGA cohort. (**K**) Differences in the PS among the MSI statuses in the TCGA cohort. (**L**) Differences in the TMB among the low- and high-PS subgroups in the TCGA cohort. (**M**) Correlation analysis of the TMB and PS in the TCGA cohort.

**Figure 8:**
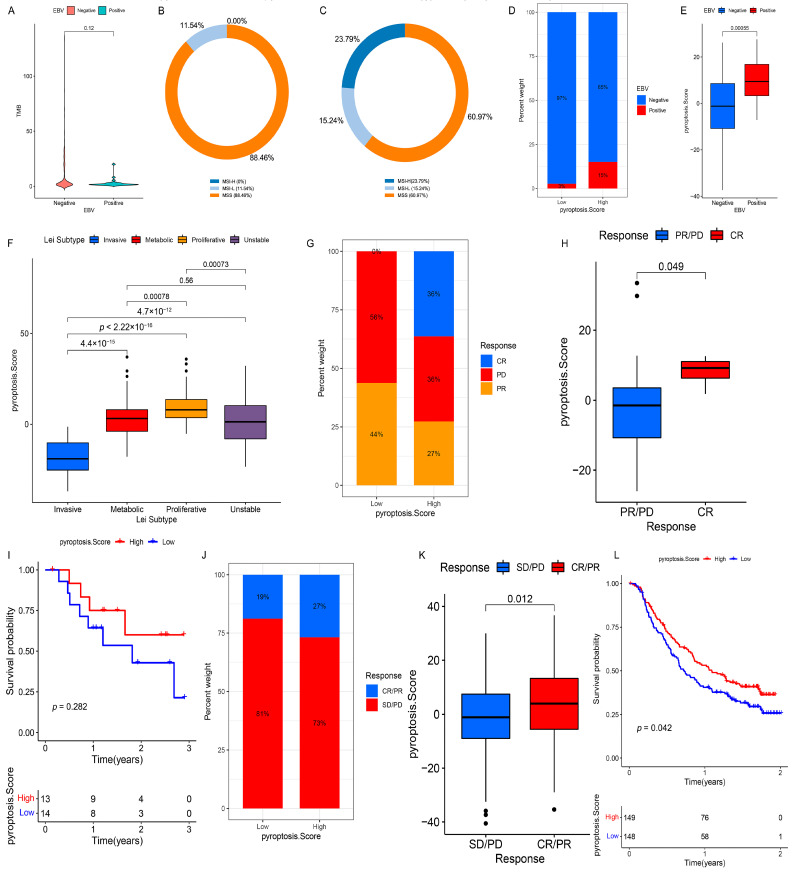
Clinical relevance of the PS and its role in predicting anti-PD-1/L1 immunotherapy. (**A**) Differences in the TMB among patients with different EBV infection statuses in the TCGA cohort. (**B**,**C**) The proportion of MSI-H, MSI-L, or MSS subtypes in the EBV-positive group (**B**) and EBV-negative group (**C**) in the TCGA cohort. (**D**) The fraction of patients with different *H. pylori* infection statuses in the low- or high-PS subgroups in the TCGA cohort. (**E**) Differences in the PS among patients with different *H. pylori* infection statuses in the TCGA cohort. (**F**) Differences in the PS among patients with Lei subtypes in the GSE15459 and GSE34942 datasets. (**G**) The fraction of patients with a clinical response to anti-PD-1 immunotherapy in the low- or high-PS subgroups in the GSE78220 dataset. CR vs. PR/PD: 36% vs. 64% in the high-PS group and 0% vs. 100% in the low-PS group. (**H**) Differences of the PS in distinct anti-PD-1 clinical response subgroups in the GSE78220 dataset. (**I**) Survival analysis for the low- and high-PS subgroups in anti-PD-1 immunotherapy using Kaplan–Meier curves (GSE78220 dataset: *p* = 0.282, Log-rank test). (**J**) The fraction of patients with a clinical response to anti-PD-L1 immunotherapy in the low- or high-PS subgroups in the IMvigor210 cohort. CR/PR vs. SD/PD: 27% vs. 73% in the high-PS group and 19% vs. 81% in the low-PS group. (**K**) Differences of the PS in distinct anti-PD-L1 clinical response subgroups in the IMvigor210 cohort. (**L**) Survival analysis for the low- and high-PS subgroups in anti-PD-L1 immunotherapy using Kaplan–Meier curves (IMvigor210 cohort: *p* = 0.042, Log-rank test). CR, complete response; PR, partial response; SD, stable disease; PD, progressive disease.

On the basis of the existing GC subtypes, we observed that the Lauren subtypes crossed the ACRG subtypes (Figure 7A), TCGA subtypes (Figure S11A), and Lei subtypes (Figure S11D) (e.g., the diffuse subtype was almost evenly distributed among the four ACRG subtypes). Although our results confirmed the role of the pyroptosis patterns in the clinical relevance and shaping different TME landscapes, as with the overlap between the existing GC subtypes, there was also a cross-correlation between the three distinct pyroptosis patterns and the existing GC subtypes (ACRG cohort: Figure 4C), which indicated that the qualitative stratification based on the patient population was defective and could not accurately predict the pyroptosis pattern and the existing subtypes in individuals. To optimize the prediction performance of the pyroptosis patterns and improve the common defects of the existing GC typing, we quantified the pyroptosis patterns on the basis of the expression of the prognostic PPRSGs (Table S6), defined as the PS. Afterwards, the diffuse subtype (Lauren typing), EMT subtype (ACRG typing), CIN/GS subtypes (TCGA typing), and invasive subtype (Lei typing) with survival weaknesses were found to be significantly enriched in the low- and middle-PS subgroups, whereas the intestinal subtype (Lauren typing), MSI cohort subtype (ACRG typing), MSI/EBV subtypes (TCGA typing), and proliferative subtype (Lei typing) with survival advantages were remarkably concentrated in the high-PS subgroup (ACRG cohort: Figure 7B; TCGA cohort: Figure S11B; Lei cohort: Figure S11E). Currently, it was difficult to find a simple correlation among the existing GC types, which indicated that classification using the qualitative algorithm was considerably limited. However, on the basis of the PS, not only did we find significant differences in the PS among the three pyroptosis patterns (meta-cohort: Figure 6G; ACRG cohort: Figure S8D), but we also observed notable differences in the PS among the existing GC types (ACRG cohort: Figure 7C,D and Figure S8F,G; TCGA cohort: Figure 7I and Figure S13A,B; Lei cohort: Figure 8F). Therefore, the quantification of the pyroptosis patterns not only discovered the correlation between the existing GC typing but also laid the foundation for individual services or the identification of individual heterogeneity.

To better illustrate the PS characteristics, we examined the relationship between the PS and existing gene signatures (meta-cohort: Figure 6D,E; ACRG cohort: Figure S8A,B) and the TME cell infiltration landscapes (meta-cohort: Figure 6F; ACRG cohort: Figure S8C) through a Spearman’s analysis. We observed that low PS was associated with stromal activation (e.g., hypoxia, EMT, angiogenesis, pan-F-TBRS, MDSC, and mast cells) and oncogenic pathways (e.g., Wnt/beta-catenin, TGF-beta, notch, and hedgehog), whereas high PS was correlated with immune activation (e.g., activated CD4+ T cells, activated CD8+ T cells, type-2/17 T-helper cells, INF-alpha/gamma response, CD8+ T effector, and antigen processing machinery) and damage repair pathways (e.g., DNA repair and mismatch repair). These results demonstrated that PS could not only better evaluate the pyroptosis patterns and the existing GC subtypes of individuals but also estimate the TME cell infiltration characterization.

### 3.7. Clinical Relevance of PS in GSE62254/ACRG Cohort

Considering the prognostic value of PS in the meta-cohort (Figure 6H, *p* < 0.001), we explored the value of PS in predicting patients’ clinicopathological outcomes. According to the cutoff value of −7.66009 determined using the survminer package, the patients were divided into low- and high-PS groups; the high-PS group showed a significant survival advantage (Figure 7H, *p* < 0.001). Univariate (HR 0.968 (0.958–0.978); Figure S15A) and multivariate analyses (HR 0.964 (0.946–0.983); Figure S15B) further confirmed PS as a robust and independent protective biomarker for evaluating a patient’s prognosis. We then investigated the association between PS and GC recurrences. The PS of patients in the relapse group was significantly lower than that in the non-relapse group (Figure 7E and Figure S8H), and the PS gradually decreased with an increase in the number of relapse sites (Figure 7F). Additionally, 16 PRs (e.g., *AIM2*, *CASP1*, and *TIRAP*) were significantly downregulated and three PRs (*GPX4*, *GSDME*, and *PJVK*) were notably upregulated in the relapse group, which might be the potential mechanisms leading to a relapse (Figure S8I). Subsequently, we investigated the ability of PS characteristics to predict the efficacy of adjuvant chemotherapy in GC patients. The results showed that the predictive power of PS was independent of adjuvant chemotherapy, and the high-PS group always showed significant OS/DFS advantages irrespective of whether the patients received chemotherapy (Figure 7G and Figure S8J). By predicting the IC_50_ of 16 compounds, the sensitivity of the high-PS group to five compounds (methotrexate, mitomycin C, paclitaxel, sorafenib, and tipifarnib) was found to be significantly higher than that of the low-PS group, and the sensitivity of the high-PS group to four compounds (temsirolimus, axitinib, bleomycin, and imatinib) was lower than that of the low-PS group (Figure S9A–P). In addition, the patients with younger ages (Figure S9Q–R); negative EBV (Figure S9S–T); upregulated *MLH1* (Figure S9U–V); and advanced clinicopathologic traits (e.g., perineural invasion, lymphovascular invasion, venous invasion, positive lymph nodes located at the body/whole, and signet ring cell carcinoma) (Figures S9W–AB and S10A–E) were correlated with the low-PS group, whereas a high-PS group was related to better clinicopathological outcomes. Moreover, we found no association between *H. pylori* infection and PS (Figure S10F,G).

### 3.8. Clinical Relevance of PS in the TCGA Cohort

The OS of the high-PS group in the TCGA cohort was significantly better than that of the low-PS group (Figure S11C). Univariate (Figure S15C) and multivariate analyses (Figure S15D) revealed that PS could act as an independent prognostic biomarker in GC. Considering that PS correlated with distinct TME cell-infiltrating landscapes, we focused on the relationship between the PS and immunotherapy assessment indicators (e.g., MSI and TMB). Statistical differences in the PS were observed among MSI-H, MSI-L, and MSS, with MSI-H being the highest, MSI-L being the second, and MSS being the lowest (Figure 7J,K). The TMB of the high-PS group was significantly higher than that of the low-PS group (Figure 7L), and an extraordinary positive correlation was observed between the TMB and PS (Figure 7M). The distribution of somatic mutations also indicated that the high-PS group had more extensive TMB than the low-PS group, with a ratio of more than three times that of the most mutated genes (Figure S12A,B). In addition, for the specific altered genes in GC, the mutant type was characterized by a higher PS than that of the wild type (e.g., *ARID1A* and *PIK3CA*) (Figure S13C–G), whereas no significant differences in the PS were observed between the wild and mutant types in *TP53* and *RHOA* (Figure S13H–K). Furthermore, the survival analysis showed that a higher TMB predicted a better OS, and a joint analysis of the PS and TMB further confirmed this outcome (Figure S14A,B). These results indirectly indicate that PS is a sensitive indicator to guide clinical immunotherapy. Several studies have revealed that patients with EBV-positive GC respond to anti-PD-1/L1 antibodies despite the relatively low MSI or TMB [26,27]. Similarly, we found no significant difference in the TMB between the EBV-positive and EBV-negative groups (Figure 8A), and the proportions of patients with MSI-H in these groups were 0.00% (EBV-positive group) and 23.79% (EBV-negative group), respectively (Figure 8B,C). However, the EBV-positive patients were more markedly correlated with a higher PS than the EBV-negative patients (Figure 8D,E), which implies that PS is a more effective biomarker for the prediction of immunotherapeutic efficacy in GC patients than MSI and TMB. Further research revealed that TMB was positively correlated with *CASP3* and *CASP6* but negatively correlated with *PLCG1* and *GSDME* (Figure S14C). Eleven PRs (e.g., *AIM2* and *CASP1*) were highly expressed in patients belonging to the MSI subtypes, whereas *ELANE*, *PLCG1*, *PRKACA*, and *GSDME* were significantly downregulated (Figure S14D). Twelve PRs (e.g., *CASP4* and *IL18*) were more upregulated in the EBV-positive patients than in the EBV-negative patients, whereas five PRs (e.g., *ELANE* and *PJVK*) were significantly downregulated (Figure S14E). These results suggest that TMB, MSI, and EBV mediate distinct pyroptosis patterns by changing the transcription levels of PRs, which contributes to our understanding of the mechanisms of the pyroptosis patterns in different tumors. Next, we compared the PS of patients from different regions or nationalities. The PS of Korean patients was significantly higher than that of patients from Russia, Vietnam, Ukraine, and the US, while no significant difference was observed among the PS of patients from other countries (Figure S13L,M). 

### 3.9. Validation of PS in Each GC Cohort and Pan-Cancer

Although we observed a remarkable prognostic value of PS in the meta-cohort, ACRG cohort, and TCGA cohort, we explored its prognostic significance in other independent GC cohorts to verify its stability (Lei cohort: Figure S11F; GSE84437: Figure S16A; GSE15459: Figure S16B; GSE57303: Figure S16C). Subsequently, we compared the areas under the ROC curves between the PS and the existing GC typing and noted that PS exhibited a better prediction performance than Lauren typing, ACRG typing, TCGA typing, and Lei typing (Figure S16D–F). Moreover, the PS model was validated in the >64 and <64 subgroups and the female and male subgroups in the ACRG cohort, and the PS had a significant predictive advantage over the existing GC typing (Figure S16G–J). Thereafter, we continued to extend the PS model to a pan-cancer analysis, and the survival analysis showed that a relatively high PS was strongly associated with favorable survival in all tumors except common tumors in women (Figure S17A–I). Furthermore, the removal of proinflammatory factors produced by pyroptosis can inhibit the growth of gynecological tumor cells and simultaneously weaken the bodily immune effects on tumor cells [11,28,29]. Therefore, pyroptosis has a dual effect of promoting and inhibiting gynecological tumors; however, the mechanism of proinflammatory factors produced by pyroptosis in gynecological tumor cells requires further investigation. In summary, we found that the integrated PR-mediated pyroptosis was more harmful than beneficial to gynecological tumors.

### 3.10. Role of PS in Predicting Anti-PD-1/L1 Immunotherapy

Considering the close connection of PS with multiple immunotherapy predictors, such as TMB and MSI, we investigated the ability of PS to predict the ICI therapeutic response in two independent immunotherapy cohorts. In both the anti-PD-1 cohort (GSE78220) and anti-PD-L1 cohort (IMvigor210), patients with a high PS showed significant therapeutic advantages (GSE78220: Figure 8G,H; IMvigor210: Figure 8J,K). Moreover, a more markedly prolonged OS to anti-PD-1/L1 immunotherapy was confirmed in patients with a high PS compared to those with a low PS (Figure 8I,L). Taken together, our findings strongly suggest that PS is remarkably correlated with the response to anti-PD-1/L1 immunotherapy and can predict patient survival outcomes.

## 4. Discussion

Inducing the immunogenic death of tumor cells is an effective strategy for building a more immunoreactive TME to transform a “cold tumor” into a “hot tumor” [30,31]. As a proinflammatory cell death form, the induction of pyroptotic cell death of tumor cells can reprogram the TME to an immunostimulatory state and alter the cytotoxic effects of cytotoxic lymphocytes on tumor cells. Extensive evidence has revealed that single PR-mediated pyroptosis has antitumor effects or promotes tumor malignant progression. Therefore, the complete recognition of the overall characteristics of the TME mediated by integrated PRs is crucial to understanding the final outcome of pyroptosis on tumor cells, which will strengthen our understanding of the TME antitumor immune response and guide effective immunotherapy.

Three distinct pyroptosis patterns were identified on the basis of 32 PRs, which had significantly different TME cell-infiltrating characteristics and were named as an immune-inflamed phenotype (pyroptosis cluster A), immune-excluded phenotype (pyroptosis cluster C), and immune-desert phenotype (pyroptosis cluster B). The immune-inflamed phenotype, also termed a “hot tumor”, is characterized by abundant immune cell infiltration, which penetrates the tumor parenchyma and acts as an antitumor agent [32]. The immune-excluded phenotype contains abundant stromal components and many immune cells; among which, the stromal components can either be localized within the tumor envelope or penetrate the tumor, whereas the immune cells stay in the stroma surrounding the tumor cell nests rather than penetrating the tumor parenchyma [33,34]. The activation of TGF-β-related pathways blocks the penetration of cytotoxic lymphocytes into the tumor parenchyma, while specific molecular inhibitors targeting TGF-β reshape the TME to an immunostimulatory state [19,35]. Therefore, patients with the immune-excluded phenotype (pyroptosis cluster C) might benefit from a combination of ICIs and TGF-β blockers. The immune-desert phenotype, also known as a “cold tumor”, is characterized by immune ignorance, a lack of activated T cells and antigen presentation, and rapid tumor growth [32,34]. Among them, rapid tumor growth can lead to the formation of hypoxic and acidic TMEs, which is unfavorable in T-cell proliferation and activation, resulting in a lower frequency of tumor-infiltrating lymphocytes and a poor prognosis [32,34]. The inhibition of cancer-dependent signaling pathways by drugs can interfere with cellular metabolic processes, which may reshape an immune-activated TME for immune-desert tumors [32,34]. Our findings were consistent with those of previous studies in terms of the TME cell-infiltrating characteristics and the activity status of the well-defined biological signatures across the three pyroptosis patterns, which confirmed the reliability of our immunophenotypic classification of the three distinct pyroptosis patterns.

The DEGs, defined as PPRSGs in this study, between the three distinct pyroptosis patterns were relevant to the immune-related pathways; this was highly similar to the findings of the functional analysis of the PRs. Similar to the cluster results for the PRs, three subtypes were identified on the basis of the PPRSGs, and the expression of the PRs and clinicopathological traits in the three subtypes were highly consistent for the three pyroptosis patterns, which proved the rationality of defining the PPRSGs. Previous studies have classified GC qualitatively by a genome-wide analysis, such as Lei typing [36], TCGA typing [37], and ACRG typing [38]. However, no simple correlation between different molecular typing classification schemes has been reported thus far; therefore, it is necessary to improve the existing molecular typing or establish new rule sets. Similarly, given the individual heterogeneity of the pyroptosis pattern, there is an urgent need to quantify the pyroptosis pattern for individual tumors. Accordingly, we set up a scoring system to evaluate individual GC pyroptosis patterns on the basis of the prognostic PPRSGs to provide the treatment strategy for individual patients more accurately. As a result, the PS of the pyroptosis patterns, which were characterized by the immune-desert phenotype, immune-excluded phenotype, and immune-inflamed phenotype, increased successively. A further analysis emphasized that the PS model could accurately predict the TME status, existing molecular subtypes, genetic variation, therapeutic response, and the clinical outcomes. We determined the robust predictive power of the PS in the ICI treatment response using two different immunotherapy cohorts (anti-PD-1/L1 immunotherapy). In particular, we found that EBV-positive patients who were sensitive to the ICI treatment and could not be identified by the TMB and MSI status [27] were significantly associated with a higher PS, suggesting that the PS model had an absolute advantage in predicting GC immunotherapy. These findings confirmed that pyroptosis patterns could be used clinically to determine patient immunophenotypes and to guide immunotherapy regimens.

In addition to elucidating the clustering results of 32 PRs, we explored the relationship between the PRs and TME cell-infiltrating characteristics and focused on the specific role of *AIM2* in regulating the tumor immunity. AIM2 may have different effects on tumor cells through distinct molecular mechanisms. AIM2 can activate inflammasomes in GC patients and act as a tumor suppressor by inducing IL-18 and IL-1β [25]. AIM2 inhibits the malignant behavior of renal cell carcinoma by inducing autophagy [39]. AIM2 attenuates hepatoma cell migration and metastasis [40]. AIM2 reduces the proliferation of colorectal cancer cells by inducing cell cycle arrest [41]. AIM2 promotes non-small-cell lung cancer cell growth through an inflammasome-dependent pathway [42]. AIM2 regulates tumor cell proliferation in glioblastoma multiforme [43]. Our analysis showed that the upregulation of *AIM2* in GC patients was closely related to immune activation, such as toxic lymphocyte infiltration and an increased number of MHC molecules. Although the molecular mechanism of AIM2 involved in regulating the biological behavior of tumor cells is complex, AIM2 has been speculated to exert an antitumor effect, mainly by activating the inflammasomes in GC patients.

The current study has several limitations. First, although we selected a catalog of 32 recognized PRs, a number of emerging PRs need to be enrolled in the model to optimize the accuracy of the pyroptosis patterns. Second, retrospective datasets were used to identify the pyroptosis patterns and construct the PS model; therefore, a prospective cohort of GC patients with immunotherapy is required to validate our findings. Third, we briefly discussed the prognostic value of the PS in pan-cancer, whereas the detailed role of pyroptosis in individual tumors needs to be explored further. Nevertheless, the PS model integrated a variety of biomarkers, including the TMB, EBV infection, MSI status, TME cell-infiltrating characteristics, stromal components, genetic variation, existing molecular subtypes, and clinicopathologic information, which could be more effective predictors of immunotherapy.

## 5. Conclusions

In conclusion, in this study, we comprehensively explored the association between pyroptosis patterns mediated by integrated PRs and TME cell-infiltrating characteristics, which demonstrated that pyroptosis was a nonnegligible factor causing the heterogeneity and complexity of TME and laid a critical foundation for understanding tumor immunoregulation. In addition, the evaluation of individual tumor pyroptosis patterns might not only improve our recognition of TME cell-infiltrating characteristics but also provide vital insights for immunotherapy.

## Data Availability

All data analyzed during this study were obtained from the GEO database (https://www.ncbi.nlm.nih.gov/geo/, accessed on 23 September 2021) and TCGA database (http://cancergenome.nih.gov/, accessed on 23 September 2021).

## Supplementary Materials

The following are available online at https://www.mdpi.com/article/10.3390/genes12101535/s1: Figure S1: Summary of this study. Figure S2: Comprehensive analysis of 33 PRs based on the TCGA cohort. Figure S3: Correlation between PR mutations and their expression. Figure S4: Unsupervised clustering of PRs in the meta-cohort, and transcriptome traits and TME characteristics in distinct pyroptosis patterns. Figure S5: Correlation between TME infiltration cells and PRs and the role of AIM2 in GC. Figure S6: Unsupervised clustering of PRs in the GSE62254/ACRG cohort and clinicopathologic traits in three pyroptosis patterns. Figure S7: Unsupervised clustering of pyroptosis pattern-related genes in the GSE62254/ACRG cohort. Figure S8: Correlation between the PS and TME status and relevant biological signatures in the GSE62254/ACRG cohort. Figure S9: Prediction of therapeutic sensitivity for multiple compounds in the GSE62254/ACRG cohort. Figure S10: Correlation between the PS and clinicopathologic traits in the GSE62254/ACRG cohort. Figure S11: Correlation between the PS and TCGA typing and Lei typing. Figure S12: Correlation between the PS and tumor somatic mutations. Figure S13: Correlation between the PS and clinicopathologic traits in the TCGA cohort. Figure S14: The effects of the TMB, microsatellite status and EBV infection on PRs. Figure S15: Independent prognostic analysis. Figure S16: Prognostic value of PS in the GC cohorts. Figure S17: Prognostic value of PS in pan-cancer. Table S1: Basic information of the six datasets enrolled in this study. Table S2: Thirty-three pyroptosis regulators collected from the literature. Table S3: Univariate analysis for the pyroptosis regulators. Table S4: The changes of the pyroptosis clusters, gene clusters, and pyroptosis score. Table S5: Differential expression analysis between pyroptosis cluster A, pyroptosis cluster B, and pyroptosis cluster C. Table S6: Univariant analysis that identified 589 prognostic pyroptosis pattern-related signature genes (PPRSGs).
